# Co-Expression of Chromatin Assembly Factor 1 Subunit A and Proliferating Cell Nuclear Antigen Is a Prognostic Biomarker of Esophageal Cancer

**DOI:** 10.3390/biomedicines11041184

**Published:** 2023-04-16

**Authors:** Bing Wen, Dan-Xia Deng, Lian-Di Liao, Zhi-Da Zhang, Ya-Qi Zheng, Ke Dong, Li-Yan Xu, En-Min Li

**Affiliations:** 1 The Key Laboratory of Molecular Biology for the High Cancer Incidence Coastal Chaoshan Area, Department of Biochemistry and Molecular Biology, Shantou University Medical College, Shantou 515041, China; 16bwen@stu.edu.cn (B.W.);; 2 Guangdong Provincial Key Laboratory of Infectious Diseases and Molecular Immunopathology, Institute of Oncologic Pathology, Shantou University Medical College, Shantou 515041, China

**Keywords:** prognostic marker, chromatin assembly factor 1 subunit A, proliferating cell nuclear antigen, DNA replication, esophageal cancer

## Abstract

(1) Background: Esophageal cancer (EC) is an important global health challenge. Due to the lack of necessary biomarkers and therapeutic targets, the survival of EC patients is poor. The EC proteomic data of 124 patients recently published by our group provides a database for research in this field. (2) Methods: Bioinformatics analysis was used to identify DNA replication and repair-related proteins in EC. Proximity ligation assay, colony formation assay, DNA fiber assay, and flow cytometry were used to study the effects of related proteins on EC cells. Kaplan–Meier survival analysis was used to evaluate the relationship between gene expression and the survival time of EC patients. (3) Results: Chromatin assembly factor 1 subunit A (CHAF1A) was highly correlated with proliferating cell nuclear antigen (PCNA) expression in EC. CHAF1A and PCNA colocalized in the nucleus of EC cells. Compared with the knockdown of CHAF1A or PCNA alone, the double knockdown of CHAF1A and PCNA could significantly inhibit EC cell proliferation. Mechanistically, CHAF1A and PCNA synergistically accelerated DNA replication and promoted S-phase progression. EC patients with high expression of both CHAF1A and PCNA had a worse survival rate. (4) Conclusion: we identify CHAF1A and PCNA as key cell cycle-related proteins leading to the malignant progression of EC, and these proteins could serve as important prognostic biomarkers and targets for EC.

## 1. Introduction

Esophageal cancer (EC) is the sixth most common cause of cancer-related death worldwide and is therefore an important global health challenge [1]. Although overall 5-year survival rates have improved from less than 5% in the 1960s to about 20% in the past decade in some European countries, the United States, and China, the outlook for EC is still less than satisfactory [2,3,4]. Today, curative treatment for EC usually involves chemotherapy or chemoradiotherapy followed by extensive surgery, often resulting in higher recurrence rates and consistently lower patient quality of life [5]. New biomarkers that can help predict treatment response and prognosis will be valuable [6,7]. Genomics has developed rapidly in the past decade. Whole genome sequencing of different cancers has described copy number variations (CNVs) and mutation frequencies of cancer-related genes, revealing the origins of different cancers [8,9]. However, genomic data alone does not always provide sufficient insight into patient prognosis or treatment [10]. Proteomics has been regarded as the next field of precision oncology after genomics and transcriptomics. Recently, we performed proteomic analyses of 124 pairs of EC tumors and corresponding adjacent non-tumor tissues, describing the abnormal changes in proteins, phosphorylation, and pathways in EC [11]. We have found that the up-regulated proteome in EC is mainly enriched in cell cycle-related pathways, including cell cycle, DNA replication, E2F targets, and G2/M checkpoints. These results suggest that the dysregulation of cell cycle-associated proteins is one of the key factors in the occurrence and development of EC.

During replication, at the center of the replication fork, three proliferating cell nuclear antigen (PCNA) monomers combine to form a sliding clamp that plays an important role in the progression of DNA replication and provides a central scaffold that allows dynamic interactions between the replication machinery and numerous additional factors [12,13]. Chromatin assembly factor-1 (CAF-1) is a heterotrimeric histone chaperone complex that consists of three subunits: chromatin assembly factor 1 subunit A (CHAF1A, also known as p150 or CAF1A), chromatin assembly factor 1 subunit B (CHAF1B, also known as p60 or CAF1B), and histone-binding protein RBBP4 (also known as p48) [14]. CAF-1 mainly binds to newly synthesized histones (H3, especially H3.1/H3.2, and H4) by interacting with PCNA and promotes their deposition at the replication fork during the S-phase. Although the expression and interactions of CHAF1A and PCNA have been studied in other cancers [15,16], their roles in EC remain unclear.

In this study, we further analyzed our EC proteome data (PXD021701) in order to find proteins closely related to the prognosis of EC patients, so as to find the key prognostic markers of EC. We found for the first time that the co-expression of CHAF1A and PCNA can be used as a good prognostic biomarker for EC patients.

## 2. Materials and Methods

### 2.1. Cell Culture

We obtained the KYSE30, KYSE50, KYSE140, KYSE150, KYSE180, KYSE200, KYSE270, KYSE410, KYSE450, KYSE510, and KYSE520 human EC cell lines that were established by Dr. Shimada Yutaka (Faculty of Medicine, Kyoto University, Kyoto, Japan) [17]. We obtained the TE1, TE3, TE5, TE7, and TE10 human EC cell lines that were established by Dr. Nishihira (Institute of Development, Aging and Cancer, Tohoku University School of Medicine, Sendai, Japan) [18]. All EC cell lines were derived from cancer tissue of patients with esophageal squamous cell carcinoma. All cells were cultured in RPMI-1640 medium supplemented with 10% fetal bovine serum (FBS), penicillin (100 mg/mL), and streptomycin (100 mg/mL) in a humidified 5% CO_2_ atmosphere at 37 °C and were tested for mycoplasma contamination using short tandem repeat (STR) analysis (IGEbio, Guangzhou, China).

### 2.2. SiRNAs and Chemical

Cells were transfected with siRNAs using Lipofectamine RNAiMAX (13778150, Thermo Fisher Scientific, Waltham, MA, USA) using standard protocols. When used in six-well plates, siRNAs were mixed with 3 µL RNAiMAX in 200 µL Opti-MEM medium to achieve a final concentration of 10 nM siRNAs. After incubating at room temperature for 10 min, the transfection mixture was added to the cultured cells. After 12 h, medium was replaced with fresh medium, and the target protein was assayed for knockdown at 48–72 h of transfection. The siRNAs targeted PCNA (siPCNA#1: 5′-GCU CCA UCC UCA AGA AGG U-3′, siPCNA#2: 5′-GCG UGA ACC UCA CCA GUA U-3′, and siPCNA#3: 5′-GCA CCA AAC CAG GAG AAA G-3′) and CHAF1A (siCHAF1A#1: 5′-CAG CCA UGG AUU GCA AAG A-3′, siCHAF1A#2: 5′-CAG AAC GAC AAG UUG GCA U-3′, and siCHAF1A#3: 5′-CUC CGC AGA AUA ACU AAG A-3′) and were purchased from GenePharma (Shanghai, China). In some experiments, three pooled siRNAs were used. Thymidine (T1895) was purchased from Sigma-Aldrich (St. Louis, MO, USA).

### 2.3. Western Blotting

Western blot analysis was performed using standard protocols, as described previously [19]. Briefly, samples were heated at 95 °C for 5–15 min in 1× sodium dodecyl sulfate (SDS) loading buffer, subjected to electrophoresis with 10% SDS-polyacrylamide gels, and transferred to polyvinylidene difluoride (PVDF) membranes. Blocking and antibody dilution were performed using 5% nonfat milk (or bovine serum albumin, BSA) in phosphate-buffered saline (PBS). After incubation with primary antibodies (Table 1) at 4 °C overnight and secondary antibodies at 25 °C for 1.5 h, the membranes were washed three times in 1× PBST (1× PBS with 0.1% Tween-20). Images were acquired using a ChemoDoc MP system (Bio-Rad, Hercules, CA, USA) with Image Lab Software.

### 2.4. Cell Fractionation

Cell fractionation experiments were performed as described previously [20], with slight modifications. KYSE30, KYSE450, or KYSE510 cells in 10 cm dishes were digested using trypsin, collected by centrifugation at 500× *g* for 5 min, washed with PBS, and lysed on ice in 600 μL of hypotonic buffer (10 mM Tris-HCl, pH 7.5, 2 mM MgCl_2_, 3 mM CaCl_2_, 320 mM sucrose, 1 mM dithiothreitol (DTT), and 0.3% NP40) supplemented with protease inhibitor cocktail (HY-K0010, MCE). After 10 min, the cells were centrifuged for 5 min at 2800× *g*, and the supernatant containing cytoplasmic proteins was collected. The pellet was washed three times in hypotonic buffer, incubated with 100 μL nuclear extraction buffer (20 mM 2-[4-(2-hydroxyethyl) piperazin-1-yl] ethanesulfonic acid (HEPES), pH 7.7, 1.5 mM MgCl_2_, 420 mM NaCl, 200 mM ethylene diamine tetraacetic acid (EDTA), 25% glycerol, and 1 mM DTT) supplemented with a protease inhibitor cocktail (HY-K0010, MCE) for 30 min at 4 °C, and then centrifuged at 8000× *g* for 15 min. The supernatant containing soluble nuclear proteins was collected and the chromatin pellet was resuspended in 1× SDS loading buffer, sonicated, and subjected to western blot analysis.

### 2.5. DNA Fiber Assay

The DNA fiber assay was performed as described previously [21]. First, KYSE30 or KYSE510 cells were incubated with 33 μΜ CldU (I7125, Sigma-Aldrich) at 37 °C for 20 min, followed by 330 μM IdU for 30 min. Labeled cells were quickly trypsinized, resuspended in ice-cold PBS at a density of approximately 1 × 10^6^ cells/mL, and spotted (2.5 μL) onto a pre-cleaned glass slide for lysis in 7.5 μL of lysis buffer (0.5% SDS in 200 mM Tris-HCl (pH 7.4) with 50 mM EDTA). After 5 min, the slides were tilted at 25 °C relative to the horizontal, and the resulting DNA spreads were air-dried and fixed in 3:1 methanol/acetic acid for 20 min. After rehydration in PBS, the samples were denatured in 2.5 M HCl for 1 h, washed with PBS, and blocked with 2% BSA in PBS (*w*/*v*) containing 0.1% Tween-20 for 40 min. Immunodetection was performed using the following primary antibodies: anti-CldU (rat monoclonal anti-5-bromo-2-deoxyuridine (BrdU)/CldU; BU1/75 ICR1, Abcam, Cambridge, UK; 1:200) and anti-IdU (mouse monoclonal anti-BrdU/IdU; clone B44, BD Biosciences, NJ, USA; 1:25). The samples were then incubated with the following secondary antibodies in a humidified chamber for 1 h at 25 °C: Alexa Fluor^®^ 647 AffiniPure donkey anti-mouse (715-605-150, Jackson ImmunoResearch, West Grove, PA, USA; 1:200) or goat anti-rat Alexa Fluor 488 (A-11006, Invitrogen, San Francisco, CA, USA; 1:200). Images were acquired using a Zeiss (Jena, Germany) LSM 800 fluorescence microscope at 40× magnification and analyzed using ImageJ software (version 1.52). A minimum of 100 individual fibers were analyzed per experiment. Statistical analyses were performed using Prism (version 8.0.2, GraphPad, Boston, MA, USA).

### 2.6. Flow Cytometry

KYSE30 or KYSE510 cells in six-well plates were trypsinized, washed once with PBS, and fixed in cold 70% ethanol at 4 °C overnight before being stored for up to one week. The cells were then washed once with PBS and incubated with propidium iodide (PI) (50 μg/mL) and RNase A (100 μg/mL) in PBS in the dark for 30 min at 37 °C. For cell cycle analysis, the cells were treated with 10 μM EdU for 30 min, trypsinized, and washed once with PBS before being fixed in cold 70% ethanol at 4 °C overnight and stored for up to one week. The samples were then analyzed using a BeyoClick™ EdU Cell Proliferation Kit with Alexa Fluor 647 (C0081L, Beyotime Biotechnology, Shanghai, China). Briefly, the cells were washed once in PBS with 2% BSA and incubated in PBS containing 2% BSA, PI (50 μg/mL), and RNase A (100 μg/mL) in the dark for 30 min at 37 °C. The cells were then selected using a flow cytometer (BD Biosciences) with side scatter (SSC) and forward scatter (FSC). DNA staining with PI reflected the cell cycle G1, S, and G2/M stages. DNA staining with EdU reflected the S-phase of the cell cycle.

### 2.7. PLA

Proximity ligation assays (PLAs) were performed using Duolink PLA technology (Sigma-Aldrich, St. Louis, MO, USA) according to the manufacturer’s instructions. Briefly, cells were washed once with 1× PBS and treated with cytoskeletal (CSK) extraction buffer [0.2% Triton X-100, 20 mM HEPES-KOH (pH 7.9), 100 mM NaCl, 3 mM MgCl_2_, 300 mM sucrose, 1 mM ethylenebis (oxyethylenenitrilo)-tetraacetic acid (EGTA)] containing 4% formaldehyde at 25 °C for 10 min. The cells were then washed three times with 1× PBS, permeabilized with 0.5% NP-40 in 1× PBS for 5 min, and blocked with 5% BSA in PBS at 25 °C for 1 h. After incubation with the indicated primary antibodies at 4 °C overnight, the cells were washed three times with 1× PBS and incubated with anti-mouse and anti-rabbit plus PLA probes (PLA kit, Sigma-Aldrich) at 37 °C for 1 h. The PLA reaction was performed using Duolink in Situ Detection Reagents (PLA kit) according to the manufacturer’s instructions. Finally, the cells were washed three times with buffer B, stained with 4′,6-diamidino-2-phenylindole (DAPI) during the second wash, and mounted with antifade mounting medium (Beyotime) on slides, coverslipped, and sealed with nail polish. Images were captured using a Zeiss LSM 800 fluorescence microscope (40×) and quantified using ImageJ software.

### 2.8. Clonogenic Assay

Clonogenic assays were performed as described previously [19]. Briefly, cells were seeded in 12-well plates and incubated for 7–14 days at 37 °C with 5% CO_2_. After washing with PBS, the cultures were fixed with methanol for 20 min and stained with crystal violet overnight. Colonies were imaged using a ChemoDoc MP system (Bio-Rad, Hercules, CA, USA) and analyzed using Image Lab (Bio-Rad, Hercules, CA, USA) and ImageJ software. Each experiment was performed in triplicate.

### 2.9. Quantification and Statistical Analysis

All statistical analyses were performed using Prism software (version 8.0.2, GraphPad). Significant differences were determined using *p*-values (* *p* < 0.05, ** *p* < 0.01). Comparisons between two groups were made using unpaired two-tailed t-tests. All plotted values represent the mean ± standard deviation (SD). Data in graphs displaying fold changes represent the mean ± SD of fold changes calculated from the mean of control samples.

## 3. Results

### 3.1. Screening Key DNA Replication and Repair Proteins in the EC Proteome

**Figure 1:**
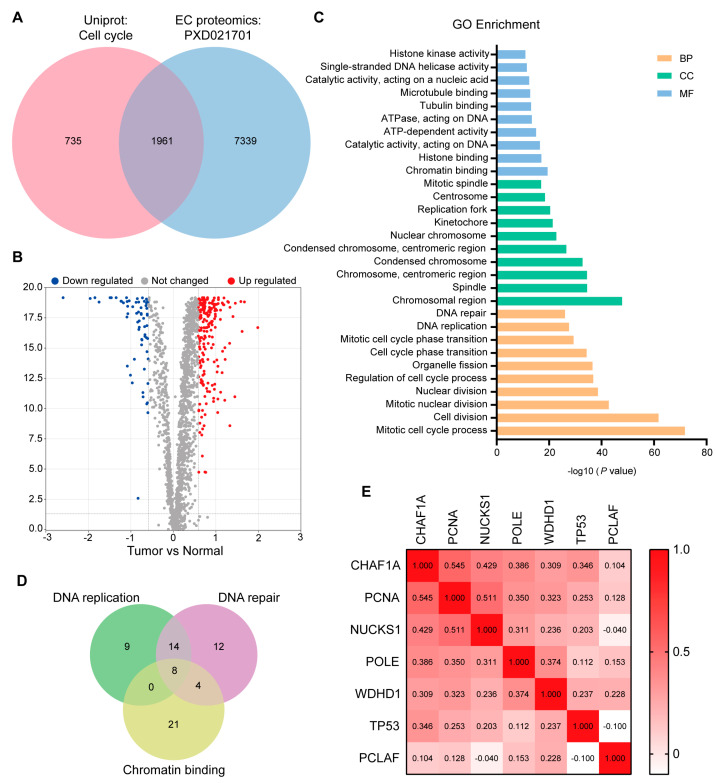
Screening of key DNA replication and DNA repair proteins by proteomic analysis. (**A**) Overlap between the “Cell Cycle” protein list in the Uniprot database and the EC proteome list. (**B**) Volcano plot showing proteins up– (red) or down–regulated (blue) in tumors. Gray dots represent other proteins. Fold change > 1.5, *p*–value < 0.01. (**C**) Gene Ontology (GO) enrichment analysis of proteins up-regulated in EC. BP: biological processes, CC: cellular components. MF: molecular functions. (**D**) Overlap between three gene subsets obtained by GO enrichment analysis: DNA replication, DNA repair, and chromatin binding. (**E**) Spearman correlation analysis was performed on seven proteins after the incomplete expression or the no significant survival analysis of eight proteins in (**D**) had been removed.

To identify key proteins related to the cell cycle in EC, we initially intersected the EC proteomic dataset (PXD021701) with the “Cell Cycle” dataset in the UniProt database (https://www.uniprot.org/, accessed on 24 September 2021). Differential analysis of the 1961 cell cycle-associated proteins enriched in our EC proteomic dataset (Figure 1A) revealed that the expression of 206 proteins was upregulated and that of 63 proteins was downregulated in tumor tissues compared to non-tumor tissues (fold change > 1.5, *p* < 0.05; Figure 1B). Rapid DNA replication and strong DNA repair are common features of malignant tumors [22]. After Gene Ontology (GO) enrichment analysis of the 206 upregulated proteins using Metascape [23] (Figure 1C), we screened key proteins related to DNA replication and repair by intersecting gene subsets associated with DNA replication, DNA repair, and chromatin binding. PCNA, WD repeat and HMG-box DNA binding protein 1 (WDHD1), nuclear casein kinase and cyclin cyclin-dependent kinase substrate 1 (NUCKS1), tumor protein p53 (TP53), CHAF1B, DNA polymerase epsilon catalytic subunit (POLE), CHAF1A, and PCNA clamp associated factor (PCLAF) were present in all three subsets (Figure 1D). There was no significant correlation between CHAF1B expression and the survival time of patients with EC, and thus, CHAF1B was also excluded from further analyses. However, the remaining proteins, including CHAF1A, PCNA, NUCKSL1, POLE, WDHD1, TP53, and PCLAF, may play key roles in DNA replication and repair in EC. To confirm the relationship between CHAF1A, PCNA, NUCKSL1, POLE, WDHD1, TP53, and PCLAF, we conducted a correlation analysis of their expression in EC tumors and found that CHAF1A had the strongest correlation with PCNA (Figure 1E). Based on these results, we conclude that CHAF1A and PCNA expression are strongly correlated in EC tumors, and they may synergistically promote the progression of EC.

### 3.2. CHAF1A Interacts with PCNA in the Nucleus of EC Cells

**Figure 2:**
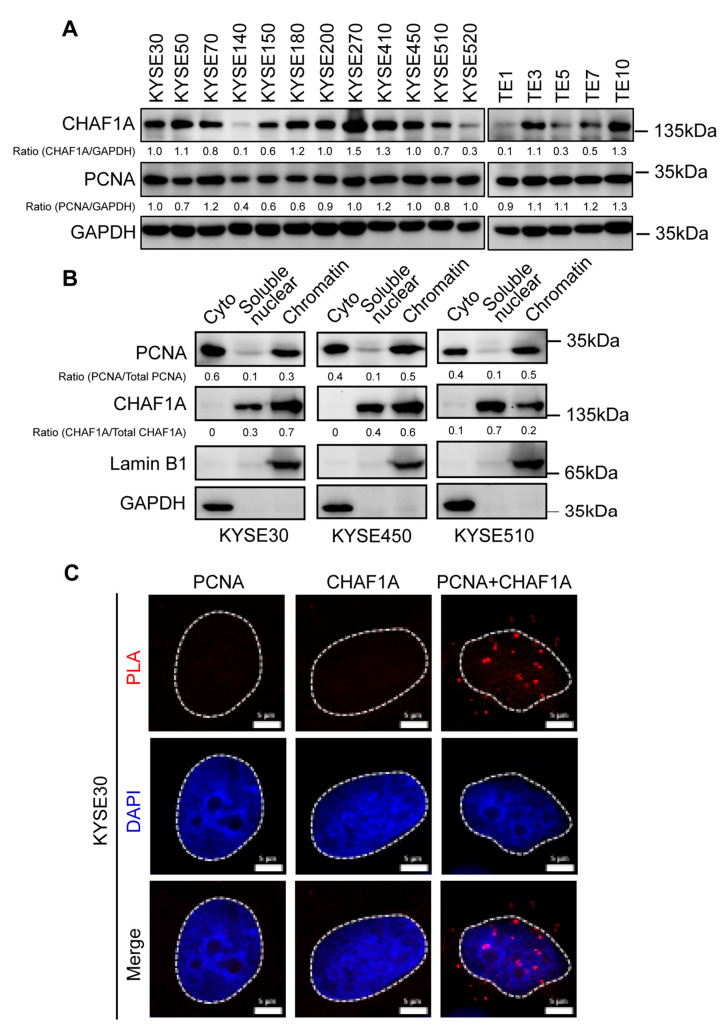
Expression, localization, and interaction of CHAF1A and PCNA in EC cells. (**A**) CHAF1A and PCNA expression in 17 EC cell lines detected by western blotting. (**B**) Cytoplasmic (Cyto), soluble nuclear, and chromatin distribution of PCNA and CHAF1A in KYSE30, KYSE450, and KYSE510 cells subjected to cell fractionation assays and western blotting. Lamin B1 was used as a chromatin marker, and GAPDH was used as a cytoplasmic marker. (**C**) Co-localization of PCNA-CHAF1A was determined using a PLA probe in KYSE30 cells. Scale bars, 5 µm.

Although the expression and interactions of CHAF1A and PCNA have been studied in other cancers [15,16], their roles in EC remain unclear. Therefore, we examined CHAF1A and PCNA expression in 17 EC cell lines and found that they were expressed in most EC cell lines (Figure 2A). Three cell lines (KYSE30, KYSE450, and KYSE510) with CHAF1A and PCNA expression were selected for cell fractionation. Interestingly, PCNA expression was mainly found in the cytoplasm and chromatin, with a low expression in soluble nuclear components, whereas CHAF1A expression was mainly found in soluble nuclear components and chromatin, with a low expression in the cytoplasm (Figure 2B). To determine the interaction between CHAF1A and PCNA in EC cells, we performed proximity ligation assays (PLAs) using KYSE30 cells and found that endogenous CHAF1A interacts with PCNA (Figure 2C). Together, these results demonstrate that CHAF1A co-localizes and interacts with PCNA in the nucleus of EC cells.

### 3.3. CHAF1A and PCNA Promote EC Cell Proliferation

**Figure 3:**
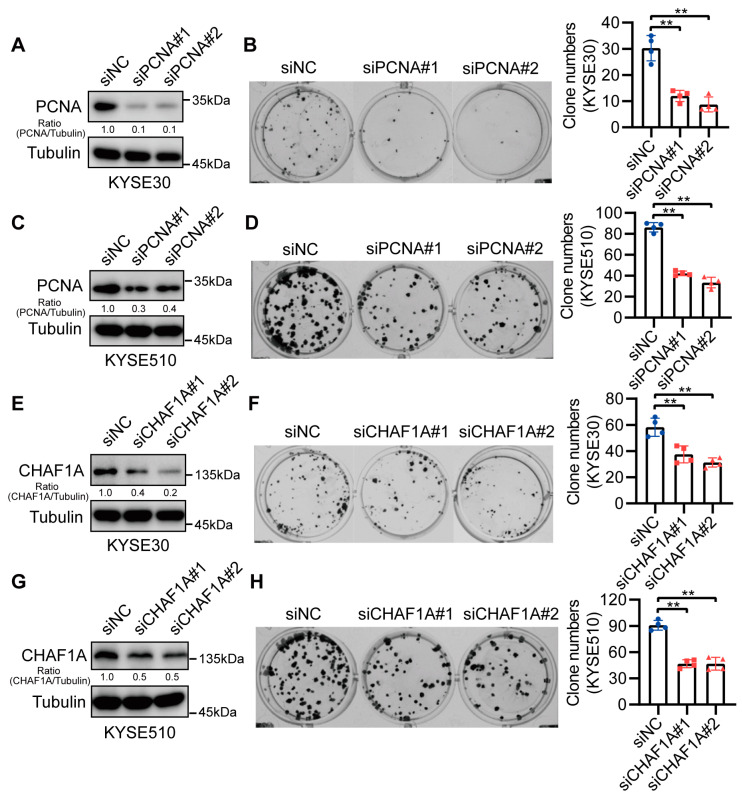
CHAF1A and PCNA knockdown reduce EC cell proliferation. (**A**,**C**,**E**,**G**) Western blot analysis of PCNA or CHAF1A knockdown in KYSE30 and KYSE510 cell lines. (**B**,**D**) Representative images (left) and colony quantification (n = 4; right) of KYSE30 cells (**B**) and KYSE510 cells (**D**) with PCNA knockdown. (**F**,**H**) Representative images (left) and colony quantification (n = 4; right) of KYSE30 cells (**F**) and KYSE510 cells (**H**) with CHAF1A knockdown. ** *p* < 0.01.

CHAF1A has been reported to act as an oncogenic factor in many cancers [14]. Therefore, we performed clonogenic assays to elucidate the functions of CHAF1A and PCNA in ECs. The siRNA-mediated knockdown of CHAF1A and PCNA in KYSE30 and KYSE510 cells (Figure 3A,C,E,G) significantly inhibited EC cell growth (Figure 3B–F,H). These results suggest that CHAF1A and PCNA synergistically promote EC cell proliferation.

### 3.4. CHAF1A and PCNA Accelerate DNA Replication in EC Cells

**Figure 4:**
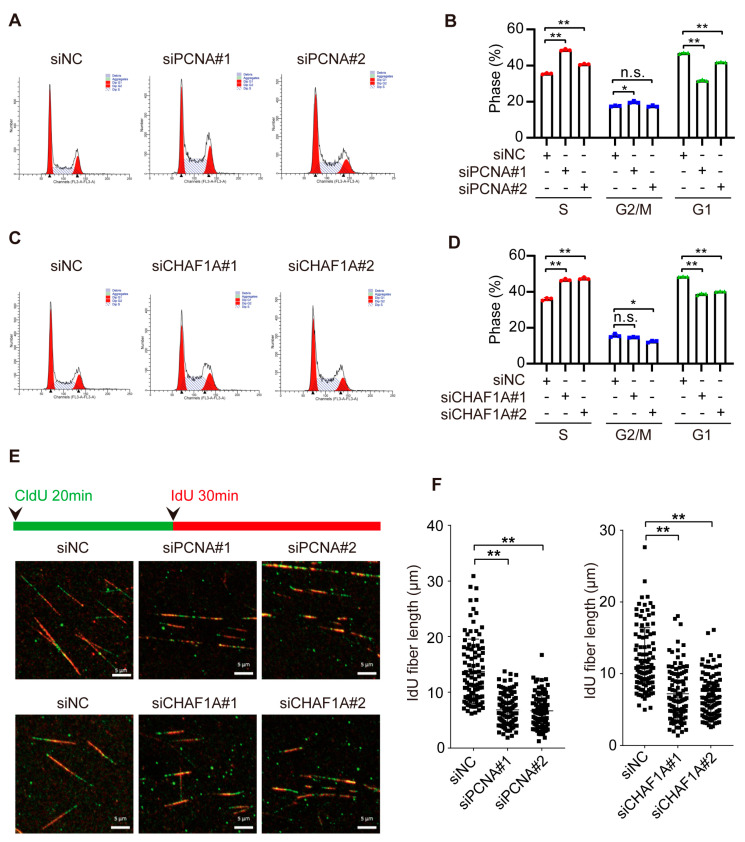
CHAF1A and PCNA facilitate DNA replication in EC cells. (**A**,**C**) Flow cytometry analysis of the cell cycle (left panel) and (**B**,**D**) S–phase quantification (n = 3; right) in KYSE30 cells transfected with siNC, siPCNA, or siCHAF1A. (**E**) Schematic of alternative 5–chloro–2–deoxyuridine (CldU)/5–iodo–2–deoxyuridine (IdU) pulse–labeling protocol to evaluate fork length in KYSE30 cells transfected with siNC, siPCNA, or siCHAF1A. (**F**) Representative images and scatterplots of IdU tract length for individual forks (n = 100). Flow cytometry analysis of the cell cycle was performed using ModFit software. * *p* < 0.05, ** *p* < 0.01, n.s.: non–significance.

To investigate whether CHAF1A and PCNA affect EC cell proliferation by influencing DNA replication, we analyzed their roles in the S-phase of cell division. Notably, knocking down CHAF1A or PCNA can increase the proportion of S phase cells and decrease the proportion of G1 phase cells, but had little effect on the proportion of G2/M phase cells (Figure 4A–D). Next, we used DNA fiber assays to directly examine the effect of PCNA or CHAF1A on DNA replication forks. Nascent DNA was sequentially labeled with the thymidine analogs 5-chloro-2-deoxyuridine (CldU) and 5-iodo-2-deoxyuridine (IdU). Interestingly, CldU- and IdU-labeled replication tracts were shorter in EC cells with PCNA or CHAF1A knockdown than in control cells (Figure 4E,F). Taken together, these results indicate that CHAF1A and PCNA promote the S phase by accelerating the DNA replication rate.

### 3.5. CHAF1A and PCNA Cooperate to Promote the S-Phase Progression of EC Cells

**Figure 5:**
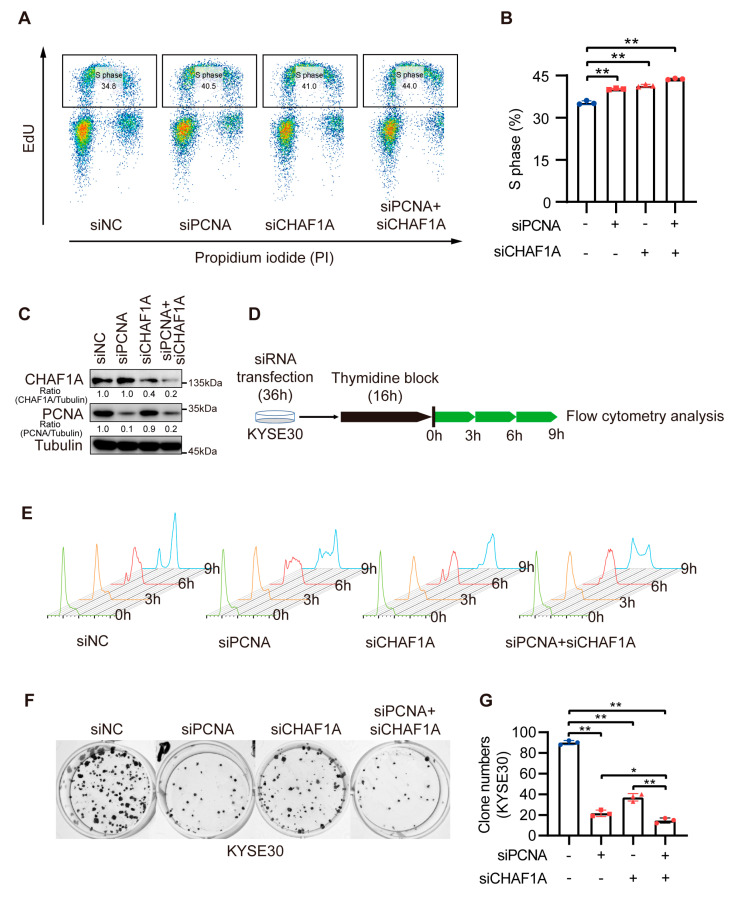
Double knockdown of CHAF1A and PCNA increases the proportion of EC cells in S–phase. (**A**,**B**) Flow cytometry analysis of EdU and propidium iodide (PI) staining in KYSE30 cells transfected with siNC, siPCNA, or siCHAF1A. The percentage of S phase cells is shown (n = 3). (**C**) Knockdown efficiency was detected using western blotting. (**D**,**E**) Schematic of cell cycle synchronization in KYSE30 transfected with siNC, siPCNA, or siCHAF1A. Flow cytometry analysis of the cell cycle was performed using FlowJo software. (**F**,**G**) Representative images (left) and colony quantification (n = 3; right) of KYSE30 cells. siRNA pools were used for single or/and double PCNA and CHAF1A knockdown. * *p* < 0.05, ** *p* < 0.01.

Because of the high correlation between CHAF1A and PCNA expression, we hypothesized that CHAF1A and PCNA promote the progression of EC through the synergistic acceleration of cancer cell DNA replication. To test this conjecture, we co-knocked down CHAF1A and PCNA in EC cells. To determine exactly how the loss of PCNA and/or CHAF1A affected the S-phase, we examined the cell cycle profiles of cells with PCNA and/or CHAF1A knockdown by flow cytometry after nascent DNA labeling with 5-ethynyl-2-deoxyuridine (EdU). Asynchronously growing PCNA or/and CHAF1A knockdown cell populations had a higher proportion of S-phase cells (EdU-positive) than the corresponding control cell populations. Moreover, double PCNA and CHAF1A knockdown had a synergistic effect on S-phase arrest in EC cells (Figure 5A,B). To further characterize the effects of PCNA and CHAF1A loss, EC cells were synchronized at the G1/S boundary, using thymidine, and then released at specific times (Figure 5C,D). Subsequent flow cytometry analysis revealed that the S-phase was longer in PCNA or CHAF1A knockdown EC cells than in WT cells (Figure 5E). Furthermore, we used clonogenic assays to demonstrate the effect of CHAF1A and PCNA co-knockdown on EC cell proliferation and showed that CHAF1A and PCNA co-knockdown significantly inhibited EC cell proliferation compared with either the control group (siNC) or single knockdown groups (siPCNA and siCHAF1A) (Figure 5F,G). Furthermore, we used clonogenic assays to demonstrate the effect of CHAF1A and PCNA co-knockdown on EC cell proliferation and showed that CHAF1A and PCNA co-knockdown significantly inhibited EC cell proliferation compared with either the control group (siNC) or single knockdown groups (siPCNA or siCHAF1A) (Figure 5F,G). Taken together, CHAF1A and PCNA synergistically enhance S-phase progression and promote EC cell proliferation.

### 3.6. High CHAF1A and PCNA Expression Are Associated with Poor Prognosis in EC

**Figure 6:**
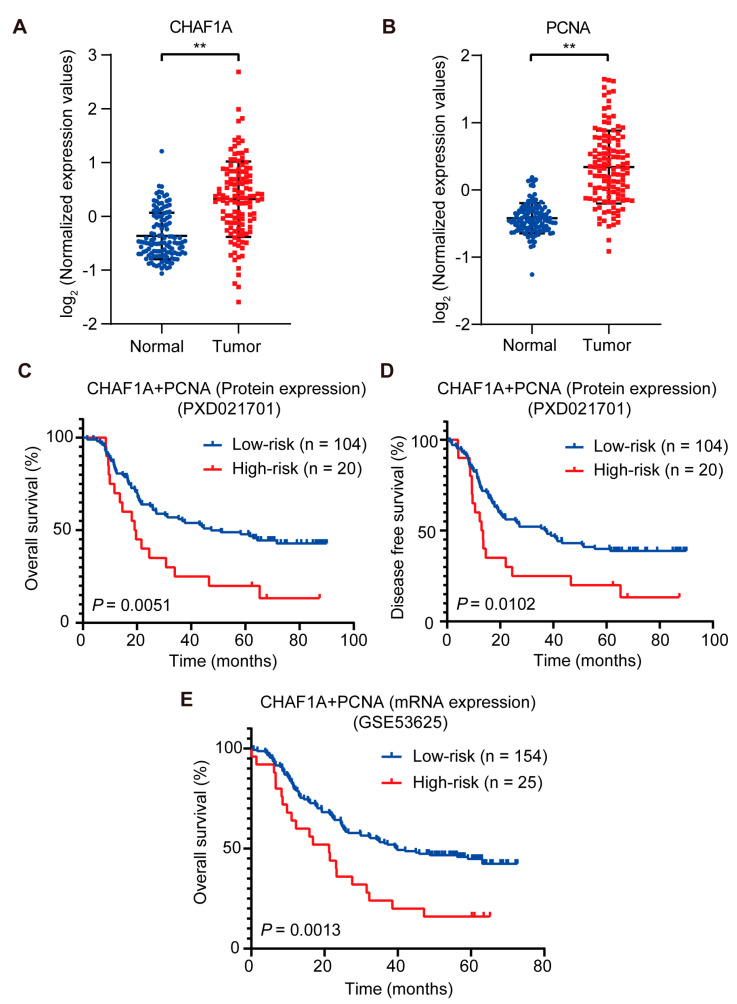
CHAF1A and PCNA are associated with poor clinical outcomes in EC. (**A**,**B**) Box plots of log_2_-transformed fold changes in PCNA and CHAF1A expression (tumor, n = 124; non–tumor, n = 124). Kaplan–Meier curves of overall survival plots (**C**) and disease–free survival plots (**D**) for high–risk (high PCNA protein expression and high CHAF1A protein expression) vs. low–risk (low PCNA protein expression or low CHAF1A protein expression) patients (dataset: PXD021701). (**E**) Kaplan–Meier curves of overall survival plots for high–risk (high PCNA mRNA expression and high CHAF1A mRNA expression) vs. low-risk (low PCNA mRNA expression or low CHAF1A mRNA expression) patients (dataset: GSE53625). ** *p* < 0.01.

To explore the clinical significance of CHAF1A and PCNA expression in EC, we analyzed the clinicopathological characteristics of 124 patients with EC in the EC proteome dataset. Notably, CHAF1A and PCNA expression were significantly upregulated in the EC tissues compared to non-tumor tissues (Figure 6A,B). The effect of CHAF1A and PCNA expression on overall survival (OS) and disease-free survival (DFS) of patients with EC was evaluated using Kaplan–Meier survival analysis. In view of the high correlation between CHAF1A and PCNA protein expression in EC tumor tissues, we next analyzed the effect of CHAF1A and PCNA protein co-expression on patient prognosis. When patients were divided into high-risk (high CHAF1A protein expression and high protein PCNA expression, n = 20) and low-risk (low CHAF1A protein expression and/or low protein PCNA expression, n = 104) groups, Kaplan–Meier survival analysis revealed that the OS and DFS were significantly higher in the low-risk group (Figure 6C,D). Then, we analyzed the effect of the co-expression of CHAF1A and PCNA mRNA on the prognosis of EC patients in the GEO database (GSE53625). The EC patients were also divided into high-risk (high CHAF1A mRNA expression and high mRNA PCNA expression, n = 25) and low-risk (low CHAF1A mRNA expression and/or low mRNA PCNA expression, n = 154) groups, Kaplan–Meier survival analysis revealed that the OS of EC patients in the high-risk group was significantly lower than that in the low-risk group (Figure 6E). Taken together, these results suggest that CHAF1A and PCNA synergistically result in the poor prognosis of patients with EC.

## 4. Discussion

In the past decade, genomic analyses of EC have been widely reported to identify the underlying genomic changes in EC development [9,24,25,26,27]. Recently, our group reported analyzing the proteome of 124 EC tissues and paired para-tumor tissues [11]. Based on the results of genomic and proteomic studies of EC, we found that the abnormal pathways in EC were mainly enriched in the cell cycle. This finding suggests that focusing on cell cycle-related proteins may help address the current problems of EC. Cell cycle control focuses on two events, replication of genomic DNA and subsequent segregation between daughter cells [28]. However, both endogenous and exogenous damage results in the activation of checkpoints that disrupt normal DNA replication [29]. In short, the cell cycle requires complete DNA replication and repair machinery. In this study, through bioinformatics analysis, we found that CHAF1A and PCNA are key cell cycle regulators when paired in EC.

The difficulty of diagnosis and treatment of EC leads to the low overall survival of EC patients, so it is urgent to find biomarkers and therapeutic targets for EC. In recent years, VEGF and cyclin D1 have been used as prognostic markers of EC [30]. Studies have shown that transmembrane proteins HER2 and PD-L1 can be used as prognostic markers of EC [31]. Ongoing clinical trials are evaluating trastuzumab, nivolumab, and pembrolizumab in combination with other treatments [32]. Cell cycle-related proteins have been found to be generally highly expressed in EC tissues [1]. However, whether cell cycle-related molecules can be used as prognostic markers in patients with EC has not been reported. In this study, we found that CHAF1A, PCNA, NUCKSL1, POLE, WDHD1, TP53, and PCLAF proteins are highly expressed in EC, and high expression of these proteins is associated with poor prognosis in EC patients (Figure 1). Unlike previous studies, we intend to use a two-protein co-expression strategy to search for prognostic biomarkers of EC. By protein expression correlation analysis, we found that CHAF1A and PCNA were the pair of proteins with the highest expression correlation among these candidates. These results suggest that CHAF1A and PCNA may have a strong synergistic effect on the malignant progression of EC. Survival analysis showed that EC patients with high co-expression of CHAF1A and PCNA had significantly shorter survival times than patients with low expression of CHAF1A and/or PCNA. These results suggest that the co-expression of CHAF1A and PCNA can be used as an indicator of malignant progression in EC patients.

In addition to identifying the co-expression of CHAF1A and PCNA as biomarkers for poor prognosis in EC patients, we found that CHAF1A and PCNA promote EC progression by accelerating DNA replication. However, there are some limitations to this study. Western blotting of 17 esophageal cancer cell lines showed that the expression of CHAF1A and PCNA were not completely consistent (Figure 2A). Interestingly, CHAF1A and PCNA are not fully colocalized on chromatin (Figure 2B), although they interact in the nucleus (Figure 2C). These results suggest that the functions of CHAF1A and PCNA may partially overlap. Knockdown of CHAF1A or PCNA significantly inhibits EC cell proliferation and DNA replication rates, which is consistent with studies in other cancers [15,16]. Surprisingly, although double knockdown of CHAF1A and PCNA had a united effect on inhibiting EC cell proliferation and DNA replication, the effect was not significant. These results suggest that CHAF1A and PCNA may have a synergistic role in DNA replication stress in addition to their key roles in normal DNA replication. Although survival analysis showed that the survival of EC patients in the high-risk group was significantly lower than that in the low-risk group, the number of patients in the high-risk group only accounted for 16% of the total (Figure 6C,D). This result indicates that the number of EC patients with high expression of CHAF1A and PCNA is relatively small, which raises new questions for the future application of this study. Moreover, the small sample size of this study is one of the limitations of this study. A larger sample of clinical data combined with immunohistochemistry can provide stronger and more favorable evidence for the subsequent application of this study.

As the most critical molecular scaffold in the process of DNA replication, PCNA interacts with numerous proteins [13]. CHAF1A is a subunit of the CAF-1 complex, which is recruited to the DNA replicating and DNA damage sites by PCNA and is responsible for histone assembly and chromatin remodeling [33]. In addition to DNA replication, CHAF1A-mediated nucleosome assembly plays a role in maintaining gene silencing in embryonic stem cells [15] and immune cells [34]. In any case, CHAF1A and PCNA-mediated nucleosome assembly during DNA replication is critical for both DNA replication and gene transcriptional regulation. It has been widely reported that CHAF1A and PCNA co-regulate histone assembly to promote cancer progression. Here, we combine them and propose that the co-expression of CHAF1A and PCNA is a prognostic biomarker for esophageal cancer. Although the deeper molecular mechanism of CHAF1A and PCNA promoting EC progression remains unclear, it still provides important evidence for their development and use as EC prognostic biomarkers.

## 5. Conclusions

We identify CHAF1A and PCNA as key molecules for poor prognosis in EC. In addition, we found that CHAF1A and PCNA promote the proliferation of EC cells by synergistic promotion of DNA replication rate and S-phase progression in EC cells. Future studies on the molecular mechanism of CHAF1A and PCNA in the malignant progression of EC will point out the direction for prognosis and targeted therapy of EC.

## Figures and Tables

**Table 1:** Antibodies used in western blotting.

Antibodies (Dilution Factor)	Source	Identifier
Mouse monoclonal anti-PCNA (1:2000)	ZEN BIO	Cat#200947-2E1
Rabbit polyclonal anti-CHAF1A (1:1000)	Proteintech	Cat#17037-1-AP
Mouse monoclonal anti-Lamin B1 (1:10,000)	Proteintech	Cat#66095-1-Ig
HRP-conjugated monoclonal GAPDH (1:20,000)	Proteintech	Cat#HRP-60004
HRP-conjugated monoclonal alpha tubulin (1:10,000)	Proteintech	Cat#HRP-66031
Goat anti-mouse IgG-HRP (1:5000)	Santa Cruz Biotechnology	Cat#sc-2005
Goat anti-rabbit IgG-HRP (1:5000)	Santa Cruz Biotechnology	Cat#sc-2030

## Data Availability

The EC proteome data were obtained from a previous paper (doi: 10.1038/s41467-021-25202-5). The “Cell Cycle” dataset was downloaded from https://www.uniprot.org/ (accessed on 24 September 2021).

## Abbreviations

BSA: bovine serum albumin; BrdU: 5-bromo-2-deoxyuridine; CHAF1A: chromatin assembly factor 1 subunit A; CHAF1B: chromatin assembly factor 1 subunit B; CldU: 5-chloro-2-deoxyuridine; DFS: disease-free survival; EC: esophageal cancer; GEO: gene expression omnibus; IdU: 5-iodo-2-deoxyuridine; NC: negative control; NUCKS1: nuclear casein kinase and cyclin-dependent kinase substrate 1; OS: overall survival; PBS: phosphate-buffered saline; PCNA: proliferating cell nuclear antigen; PCLAF: PCNA clamp associated factor; PI: propidium iodide; PLA: proximity ligation assay; POLE: DNA polymerase epsilon catalytic subunit; siRNA: small interfering RNA; TP53: tumor protein p53; WDHD1: WD repeat and HMG-box DNA binding protein 1.
